# Language growth in children with heterogeneous language disorders: a population study

**DOI:** 10.1111/jcpp.12793

**Published:** 2017-09-18

**Authors:** Courtenay Frazier Norbury, George Vamvakas, Debbie Gooch, Gillian Baird, Tony Charman, Emily Simonoff, Andrew Pickles

**Affiliations:** ^1^ Psychology and Language Sciences University College London London UK; ^2^ Department of Psychology Royal Holloway, University of London London UK; ^3^ Department of Biostatistics Institute of Psychology, Psychiatry and Neuroscience Kings College London London UK; ^4^ Newcomen Centre Guy's and St Thomas’ NHS Trust London UK; ^5^ Department of Psychology Institute of Psychology, Psychiatry and Neuroscience Kings College London London UK; ^6^ Department of Child & Adolescent Psychiatry Institute of Psychology, Psychiatry and Neuroscience Kings College London London UK

**Keywords:** Language disorder, language trajectories, longitudinal study, comorbidity

## Abstract

**Background:**

Language development has been characterised by significant individual stability from school entry. However, the extent to which trajectories of language growth vary in children with language disorder as a function of co‐occurring developmental challenges is a question of theoretical import, with implications for service provision.

**Methods:**

SCALES employed a population‐based survey design with sample weighting procedures to estimate growth in core language skills over the first three years of school. A stratified sample (*n* = 529) received comprehensive assessment of language, nonverbal IQ, and social, emotional and behavioural difficulties at 5–6 years of age and 95% of the sample (*n* = 499) were assessed again at ages 7–8. Language growth was measured using both raw and standard scores in children with typical development, children with language disorder of unknown origin, and children with language disorders associated with a known clinical condition and/or intellectual disability.

**Results:**

Overall, language was stable at the individual level (estimated ICC = 0.95) over the first three years of school. Linear mixed effects models highlighted steady growth in language raw scores across all three groups, including those with multiple developmental challenges. There was little evidence, however, that children with language disorders were narrowing the gap with peers (*z*‐scores). Adjusted models indicated that while nonverbal ability, socioeconomic status and social, emotional and behavioural deficits predicted initial language score (intercept), none predicted language growth (slope).

**Conclusions:**

These findings corroborate previous studies suggesting stable language trajectories after ages 5–6 years, but add considerably to previous work by demonstrating similar developmental patterns in children with additional nonverbal cognitive deficits, social, emotional, and behavioural challenges, social disadvantage or clinical diagnoses.

## Introduction

Language skills are critical for academic, cognitive and socioemotional functioning (Hulme, Nash, Gooch, Lervag, & Snowling, 2015; Johnson, Beitchman, & Brownlie, 2010; Petersen et al., 2013). It is therefore not surprising that children who begin school with language impairments are at significantly increased risk for long‐term academic underachievement (Durkin, Mok, & Conti‐Ramsden, 2015; Stothard, Snowling, Bishop, Chipchase, & Kaplan, 1998), social‐emotional disorder (Yew & O'Kearney, 2013) and poorer employment outcomes (Johnson et al., 2010). Epidemiological studies estimate that approximately 7% of children starting school have clinically significant language disorders of unknown origin (Norbury et al., 2016; Tomblin et al., 1997) with an additional 2.3% experiencing language disorder as part of a pervasive neurodevelopmental condition (Norbury et al., 2016). A vital question for clinical services is the extent to which it is possible to accelerate growth in language in order to ameliorate language disorder, and the negative consequences associated with this disorder (Schmitt, Logan, Tambyraja, Farquharson, & Justice, 2017). A related question concerns the impact of additional developmental challenges on language growth and the potential for recovery, especially as the goal for many clinical and education services is to narrow the achievement gap between the most able children and their lower achieving peers (Department for Education, 2014).

These questions have been challenging to answer due to potentially paradoxical forces in child language development and disorder: stability of language in relation to peers, and individual growth in language capacity (cf. Bornstein, Hahn, Putnick, & Suwalsky, 2014).

Stability has been defined as the maintenance in the rank order of individuals within a group over time, with reference to a particular characteristic (Bornstein, 2014). Standardised scores are a useful way of looking at stability, as these explicitly describe a child's performance on a particular skill relative to peers of the same age. Stability in language, in this sense, would mean that standard scores change little over the years, such that children with low‐language scores continue to score at the bottom end of the distribution of language scores. Strong stability, however, does not mean that language is immutable to change. Language growth is easier to observe when raw scores are reported over time, because these show increases in language competence. If the rate of improvement is the same at the higher and lower ends of the distribution, standard scores will remain unchanged and language is considered ‘stable’, even if there has been progress in real terms. With regard to language disorder, interventions often aim for greater than expected language growth (i.e. bigger increases in raw scores compared to peers), in order that children with early language deficits increase standard scores such that they are ‘narrowing the gap’ with typically developing peers (Schmitt et al., 2017).

Evidence is therefore needed regarding both trajectories of language growth, *and* language stability (Bornstein et al., 2014). Narrowing the gap requires accelerated language growth in children with language deficits relative to typically developing peers. A key question is whether rate of language growth is malleable, and if so, what factors predict language growth for individual children.

### Stability in language from school entry

Converging evidence from different populations demonstrates that individual differences in language skill are stable from school entry (approximate age 5–6 years). Evidence includes population cohorts, (Bornstein et al., 2014; McKean et al., 2015), populations of low‐income families (Bornstein, Hahn, & Putnick, 2016a, 2016b), children selected as having specific language impairment from epidemiological cohorts (Beitchman et al., 1996; Tomblin, Zhang, Buckwalter, & O'Brien, 2003), clinically referred and treated cases of specific language impairment (Conti‐Ramsden, St Clair, Pickles, & Durkin, 2012; Rice & Hoffman, 2015) and children with autism spectrum disorder (Pickles, Anderson, & Lord, 2014). Across all studies, language stability is impressive, with estimates ranging from 0.72 to 0.99, even in studies spanning seven years or more. Stability does not diminish the potential for growth in language competence, which Bornstein et al. (2016b) emphasise may be responsive to change, experience or intervention. However, longitudinal investigations of children with ‘specific’ language impairment have yielded little evidence that children with language disorder are able to ‘catch‐up’ with typically developing peers, despite improvement in language competence in real terms (Beitchman et al., 1996; Conti‐Ramsden et al., 2012; Rice & Hoffman, 2015; Tomblin et al., 2003). This is in part because typical children also continue to develop language; a child's raw score on a language test could improve by 20 points, but language would still be stable if other children in the population were also improving by similar degree. Thus, despite substantial language growth in real terms, rates of growth are parallel to typically developing (TD) peers, meaning that children with language disorder continue to score at the lower end of the language distribution.

### Predictors of language growth

Language abilities in both typical and atypical populations tend to be less stable before school entry (Bornstein et al., 2016a, 2016b; Pickles et al., 2014) and prediction from some language measures, such as vocabulary, prior to age three may be too unreliable to be clinically useful (Duff, Reen, Plunkett, & Nation, 2015). Identification of additional factors that improve prediction of language growth is therefore needed to effectively target early intervention resources. McKean et al. (2015) investigated a population cohort of children from the ages of 4–7 years, using standard scores from an omnibus measure of language competence. Twenty‐two variables, categorised by the extent to which they may be amenable to intervention, were analysed as potential predictors of language growth. While many of these variables predicted initial language scores, only five predicted growth. Exposure to English as an additional language, poor ratings of prosocial function, and fewer than 10 children's books at home were all associated with accelerated rates of growth. Watching more than three hours of television per day and reduced frequency of shared book reading were associated with slower rates of language growth. The direction of some effects is counter‐intuitive and the study did not distinguish language ability groups within the cohort; it is therefore possible that such relationships vary according to initial language status. Bornstein et al., (2016b) argued that those children who start school with impoverished language abilities may have more limited resources with which to catch‐up to peers, yielding slower rates of language growth and greater stability over time. The authors tested this hypothesis and the influence of five co‐variates known to associate with language development: nonverbal IQ, child positive social interactions, family home environment, maternal language and maternal education. Stability estimates did not differ between high‐ and low‐language ability groups from 5 to 11 years of age, and there was no evidence of greater stability in the low‐language group, indicating that similar processes maintain core language skills across the ability range. While all covariates were associated with language (i.e. those with language deficits tended to have lower scores on all covariates relative to high‐ability peers at all time points), they made little difference to estimates of stability. Neither study found that a child's nonverbal cognitive ability or broader behavioural problems contributed to stability. This is critical as it has been suggested that additional cognitive deficits may limit potential for resolution of early language deficits (Bishop & Edmundson, 1987) or response to language intervention (cf. Bowyer‐Crane, Duff, Hulme, & Snowling, 2011).

### Language disorder and the potential to narrow the gap

Epidemiological studies have demonstrated that language trajectories of children with specific language impairment (SLI), who are selected to have nonverbal cognitive abilities within the normal range, are parallel to that of typically developing peers (the ‘tracking hypothesis’; Law, Tomblin, & Zhang, 2008), with the initial mean difference between the two groups at age five still evident in adolescence (Beitchman et al., 2008; Tomblin & Nippold, 2014). Studies of clinically referred cohorts have not consistently included a typically developing comparison group. Those that have also report similar trajectories for children with and without language disorder. When typically developing comparison groups are not available for longitudinal study, latent growth profiling has been used to identify distinct developmental trajectories within clinical cohorts. In general, latent profiles map onto initial severity of language function, with all groups tracking in parallel, though there may be specific deficit patterns in the middle groups (Conti‐Ramsden et al., 2012; Tambyraja, Schmitt, Farquharson, & Justice, 2015). For example, Conti‐Ramsden et al. (2012) examined growth trajectories of both verbal and nonverbal skills in children educated in specialist language units at age seven. Six latent growth profiles were observed, and language profiles were more stable than nonverbal profiles. Despite differences in nonverbal cognitive ability and highly variable educational placements after age seven, all language profile groups tracked in parallel, with little evidence that those with the most severe initial language deficits were catching up with more able peers. In fact, deceleration of language growth with the onset of puberty has been documented, leading to a wider gap between young people with a history of language impairment and their typically developing peers in early adulthood (Rice & Hoffman, 2015).

All of these studies have excluded children with complex neurodevelopmental disorders or intellectual disabilities at intake. Potentially, children with clinical conditions characterised by multiple cognitive, social and behavioural deficits will demonstrate greater stability in language function and slower rates of language growth. This could reflect biological constraints on language learning, yielding fewer resources to compensate for language weaknesses. In addition, such complex language disorders may also shape later opportunities for language learning, through reduced participation in social interaction, differences in literacy attainment and special educational adjustments (cf. Bornstein et al., 2016a). These experiential differences could lead to a widening gap between children with language disorder and typically developing peers over time. Direct comparisons between studies are limited by differences in diagnostic inclusion and exclusion criteria, sampling methods and the variety of language measures used to estimate language progress. However, Pickles et al. (2014) reported similar parallel rates of language growth in children with autism spectrum disorder. Parent report of language ability was assessed at multiple points between the ages of 2 and 19 years. As with language disorder, growth in language was stable from age 6, with groups identified by initial severity and tracking in parallel across the school years. Any ‘catch‐up’ was observed between the ages of two and six, with no indication of accelerated language growth after this point (cf. Rice & Hoffman, 2015). Pickles et al. (2014) did not include a typical comparison group, however, so rate of language growth in relation to neurotypical peers is currently unclear.

### The current study

The current study provides a unique opportunity to investigate language growth and stability in a population cohort that includes children with varying degrees of verbal and nonverbal cognitive ability and a wide range of additional diagnoses. We employed linear mixed effects models to consider the influence of child (nonverbal IQ, clinical diagnosis, social, emotional and behavioural problems) and environmental (socioeconomic disadvantage) factors on language change in a robust, omnibus measure of language. Based on the extant literature, we expected children with language disorders of unknown origin to demonstrate parallel rates of language growth relative to typically developing peers. Our predictions regarding children with additional clinical concerns were more guarded, but we anticipated a slower rate of growth in raw scores and a widening gap with typical peers evident in age‐adjusted *z*‐scores (cf. Bishop & Edmundson, 1987). The current study provides a unique opportunity to assess language growth using the same measures across the first four years of mainstream education provision, and to explicitly compare children with ‘specific’ language disorder and those for whom language deficits are part of a more pervasive developmental condition.

## Methods

### Participants

**Figure 1:**
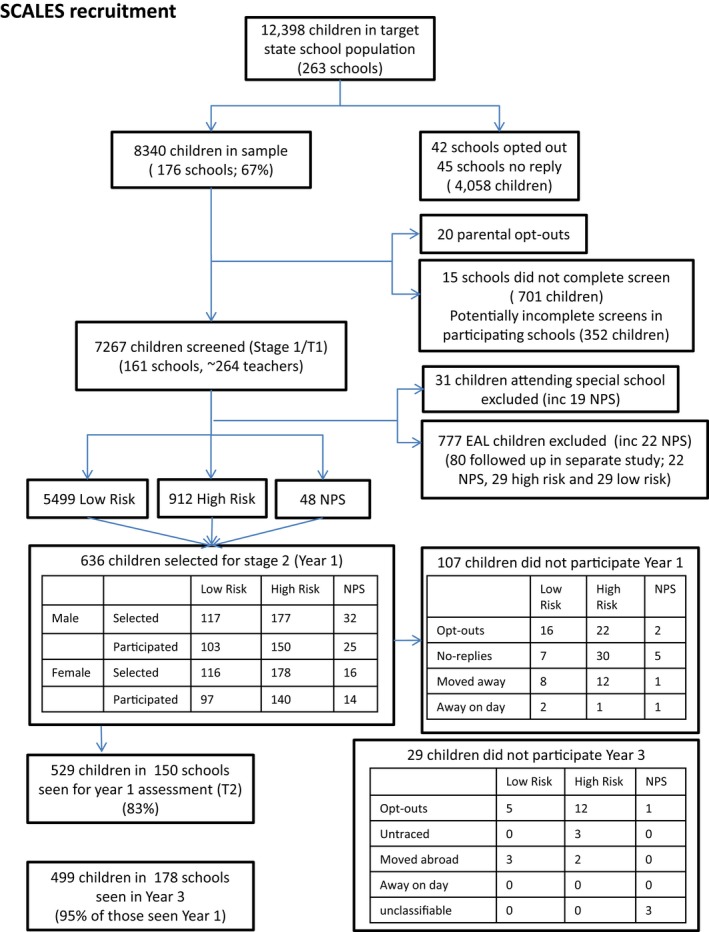
Flow chart of participation from screen to Year 3

The Surrey Communication and Language in Education Study (SCALES) used a two‐phase design (Norbury et al., 2016). In the first phase, reception (kindergarten) class teachers in 263 state‐funded primary schools were invited to complete the Children's Communication Checklist‐Short (CCC‐S, Bishop & Norbury, unpublished), a 13‐item checklist measuring language and communication skills in everyday contexts (maximum score = 39). Data were obtained between May‐July 2012 for 7,267 children (aged 57–70 months) (response rate: 61% of all eligible schools and 59% of all eligible children, Figure 1). Income Deprivation Affecting Children Index scores obtained from home postcodes provided a measure of socioeconomic status (McLennan, Barnes, Davies, Garratt, & Dibben, 2011). Index scores reflect rankings of individual neighbourhoods on the basis of local employment and receipt of means tested benefits, with rank scores in England range from 1 (most deprived neighbourhood) to 32,844 (mean rank score for all of England = 16,241), and in this sample scores ranged from 731 (most deprived neighbourhood) to 32,474 (most affluent neighbourhood) (SCALES mean = 21,592, *SD* = 7,830).

In the second phase, a subsample was selected for in‐depth assessment in Year 1 (ages 5–6 years) and Year 3 (ages 7–8 years) using stratified random sampling. Initial strata identified children who were reported as having ‘no phrase speech’ (NPS, *n* = 89, 1.2%), those attending special schools for severe learning disabilities (*n* = 31, including 19 NPS, 0.4%) and those for whom English was an additional language (*n* = 782, 10.7%, including 27 NPS). No Phrase Speech (NPS) was recorded when teachers provided a negative response to the question ‘is the child combining words into phrases or sentences?’ Children in special schools at study intake were excluded, while children with English as an additional language were invited to a different study and their data are not reported here (Whiteside, Gooch, & Norbury, 2017). All additional children with NPS (*n* = 48) were invited for in‐depth assessment.

For remaining monolingual children (*n* = 6,411), cut‐off scores on the CCC‐S were derived separately for each of three age‐groups (autumn, spring and summer) to identify sex‐specific strata of boys (13.9%) and girls (14.8%) with teacher ratings of poor language (defined as 86th centile for sex and age group). In total, 636 children (including 48 NPS) were invited to participate, with a higher sampling fraction for high‐risk children (40.5% for boys, 37.5% for girls) versus low‐risk children (4.3% for boys, 4.2% for girls). In Year 1, 529 monolingual children (83% of invited cohort) were assessed in detail. In Year 3, 499 monolingual children (95% of assessed cohort) were seen for follow‐up assessment (see Figure 1). Participant demographics are presented in Table 1.

**Table 1:** Cohort demographics. Estimated means (95% CI) reported for IDACI rank (a measure of socioeconomic status) and SDQ Total difficulties scores, and estimated percentage of males using population weights. Percentages of children in education provision or referred to speech‐language therapy are based on raw counts

	Typically developing	LD‐only	LD‐plus diagnosis
Percent male	48.43 (41.76, 55.16)	55.54 (36.71, 72.90)	75.80 (56.90, 88.15)
IDACI rank score^a^	23,704 (22,731, 24,677)	15,682 (12,881, 18,484)	20,477 (15,389, 25,565)
SDQ: Total Difficulties score^b^	5.02 (4.38, 5.65)	7.21 (5.78, 8.64)	11.94 (7.34, 16.55)
Percent with Statement of Special Educational Need	0.79	8.14	50.00
Percent in special school or unit	0	0	39.02%
Percent referred to speech‐language therapy services	22.57%	54.65%	84.09%

a Mean values for children with LD‐only differ significantly, *t*(440) = 5.31, *p* < .001. There are no other significant group differences.

b Both LD‐only and LD‐plus groups have significantly increased symptom severity scores relative to TD peers: *t*(440) = 2.76, *p* = .006 and *t*(399) = 2.93, *p* = .004 respectively.

### Consent procedures

Consent procedures and study protocol were developed in consultation with Surrey County Council and approved by the Research Ethics Committee at Royal Holloway, University of London. Opt‐out consent was adopted for the first phase as data could be provided anonymously to the research team; 20 families opted‐out. In the second phase, written, informed consent for two episodes of direct assessment was obtained from the parents or legal guardians of all participants. Prior to assessment in Year 3, families received additional information sheet and the option to withdraw from the study; 18 families withdrew consent, five moved abroad, three could not be contacted and three provided insufficient data on the day of testing for diagnostic classification. Of the 29 children (19 males) not included in follow‐up, 22 had been classified as ‘typically developing’ in Year 1 and had no evidence of language, learning or behavioural difficulties.

### Assessment

#### Nonverbal ability

Nonverbal IQ was measured using block design and matrix reasoning subtests from the *Wechsler Preschool and Primary Scales of Intelligence* (WPPSI‐3rd UK edition; Wechsler, 2003) in Year 1 (ages 5–6) and the *Wechsler Intelligence Scales for Children* (WISC 4th UK edition; Wechsler, 2004) in Year 3 (ages 7–8). Raw scores were converted to *z*‐scores using the current population sample (see below).

#### Language composites

Assessment closely followed procedures which have informed DSM‐5 diagnostic criteria and which allow direct comparison with previous epidemiological studies (Tomblin et al., 1997). Language composites were derived from six individual tests:

##### Receptive and expressive one‐word picture vocabulary tests (R/EOWPT; Brownell, 2010)

The vocabulary composite comprised word‐to‐picture matching and picture naming tests respectively. Test–retest reliability is 0.97 for both measures and internal consistency for ages 5‐ to 8‐years is excellent (Cronbach's *α *= .94–.97).

##### Test for reception of grammar (short form) (TROG; Bishop, 2003)

Forty of the original 80 sentence‐to‐picture matching items were included, with excellent agreement between short and long forms in pilot testing, *r*(17) = .88.

##### School‐age sentence imitation test‐32 items (SASIT‐32; Marinis, Chiat, Armon‐Lotem, Piper, & Roy, 2011)

Children repeated 32 sentences of increasing length and grammatical complexity and accuracy recorded. Test–retest reliability is excellent, 0.98 (Chiat & Roy, 2013). The TROG and the SASIT‐32 formed the grammar composite.

##### Assessment of comprehension and expression: narrative retelling subtest (ACE‐Recall; Adams, Cooke, Crutchley, Hesketh, & Reeves, 2001)

Children listened to a brief narrative that was prerecorded by a female, native speaker of British English and played over headphones while the accompanying pictures were displayed on a laptop computer. Children were asked to retell the story while the pictures presented on the screen. Stories were audio recorded and scored off‐line for the number of key information units recalled (range 0–35). Internal consistency is adequate (Cronbach's *α *= .73) for children aged 6‐ to 11‐years.

##### Narrative comprehension (ACE‐Comp)

Twelve bespoke comprehension questions (six literal and six inference questions) followed the narrative recall. Reponses were scored 0 for no response/incorrect answer, 1 point for partially correct response and 2 points for correct responses (maximum score = 24). All scoring was done by consensus to ensure rater consistency. These two measures formed the narrative composite.

The Expressive language composite comprised the EOWPVT, SASIT‐32 and ACE‐Recall tests, while the Receptive language composite comprised the ROWPVT, TROG and ACE‐Comp tests. In addition, a Total language composite was formed by averaging the *z*‐scores of all six direct measures. Language Disorder‐only (LD‐only) was defined as scores of −1.5*SD* or below on two out of five language composites in the absence of intellectual disability (*z*‐scores above −2*SD* on nonverbal composite) and/or existing medical diagnosis. Children designated Language Disorder‐plus (LD‐plus) met the same language criteria, but were also reported to have an existing medical diagnosis and/or nonverbal ability scores (*z*‐scores) of more than −2*SD*. The estimated means and standard deviations for all language and nonverbal ability composite scores at Year 1 and Year 3 are reported by group classification at Year 1 in Table 2. Intraclass correlations for language and nonverbal IQ composites are reported in Table 3.

**Table 2:** Estimated means (95% confidence intervals) using probability weights for key measures by language group (based at Year 1 group classification) when tested in Year 1 of school and again at Year 3. Numbers are reported the numbers of participants assessed per group prior to adjustment by probability weights. All measures are reported as *z*‐scores (see Table S1 for raw language scores)

	Typical develop (TD)		Language disorder ‐(no known diagnosis) (LD‐only)		Language disorder (known diagnosis/intellectual disability) (LD‐plus)	
Measure	Year 1 *N* = 389	Year 3 *N* = 359	Year 1 *N* = 86	Year 3 *N* = 82	Year 1 *N* = 45	Year 3 *N* = 42
Age (months)	71.66 (71.00, 72.32)	95.27 (94.67, 95.86)	71.99 (70.69, 73.30)	95.96 (94.45, 97.47)	73.95 (72.73, 75.16)	95.23 (92.79, 97.64)
Nonverbal IQ	0.13 (0.01, 0.26)	0.15 (0.03, 0.27)	−0.72 (−0.94, −0.50)	−0.87 (−1.19, −0.55)	−1.66 (−2.10, −1.23)	−1.39 (−2.21, −0.58)
Vocabulary	0.19 (0.07, 0.31)	0.19 (0.07, 0.30)	−1.55 (−1.77, −1.34)	−1.32 (−1.59, −1.06)	−1.83 (−2.11, −1.54)	−1.92 (−2.55, −1.29)
Grammar	0.16 (0.03, 0.28)	0.20 (0.08, 0.31)	−1.43 (−1.61, −1.26)	−1.35 (−1.61, −1.09)	−1.58 (−2.20, −0.96)	−1.48 (−2.36, −0.61)
Narrative	0.14 (0.02, 0.27)	0.19 (0.07, 0.30)	−1.41 (−1.67, −1.14)	−1.10 (−1.41, −0.79)	−2.11 (−2.29, −1.93)	−1.65 (−1.94, −1.36)
Receptive	0.18 (0.06, 0.30)	0.20 (0.08, 0.31)	−1.51 (−1.69, −1.32)	−1.28 (−1.52, −1.04)	−2.07 (−2.25, −1.89)	−1.85 (−2.30, −1.40)
Expressive	0.17 (0.04, 0.30)	0.21 (0.10, 0.31)	−1.61 (−1.81, −1.41)	−1.43 (−1.70, −1.15)	−1.81 (−2.16, −1.46)	−1.68 (−2.40, −0.96)
Total language	0.19 (0.07, 0.31)	0.22 (0.11, 0.32)	−1.68 (−1.85, −1.52)	−1.45 (−1.72, −1.19)	−2.11 (−2.40, −1.81)	−1.93 (−2.57, −1.28)

**Table 3:** Estimated intraclass correlation coefficients across entire cohort (estimated *N* = 6,464) between the same language and nonverbal measures at Year 1 and Year 3

Measure	Intraclass correlation (Year 1–Year 3)	95% Confidence interval
1 Nonverbal IQ	0.834	0.800, 0.863
2 Vocabulary	0.912	0.893, 0.927
3 Grammar	0.909	0.890, 0.925
4 Narrative	0.817	0.783, 0.847
5 Receptive	0.894	0.870, 0.915
6 Expressive	0.916	0.895, 0.932
7 Total language	0.940	0.926, 0.951

Note: All tests were the same except nonverbal ability, in which matrix reasoning from the WPPSI was used at Year 1 and from the WISC at Year 3

#### Clinical diagnosis and social, emotional and behavioural symptoms

Diagnostic information was elicited from teachers during the first on‐line questionnaire phase and from parents and/or the school special educational needs co‐ordinator (SENCO) during the second phase assessment using a checklist of possible diagnoses (Table 4). Teachers also completed the *Strengths and Difficulties Questionnaire* (SDQ: Goodman, 1997) at study intake, a well‐validated questionnaire rating social, emotional and behavioural development. The Total Difficulties score was used as a predictor of language growth.

**Table 4:** Unweighted frequencies of children with known clinical diagnoses or intellectual impairment as reported by teachers and/or parents in Year 1 and Year 3

Primary medical diagnosis	Unweighted frequency	*N* males
Hearing impairment	6	4
Visual Impairment	5	2
ASD	33	29
Epilepsy	3	3
Head injury/Neurological impairment	3	1
Cerebral Palsy	1	1
Down syndrome	2	1
Noonan syndrome	1	1
Neurofibromatosis	1	1
Other chromosomal anomaly	2	2
Intellectual Disability (≤−2*SD* on NVIQ tests)	37	23
Total	94	68

#### Educational and clinical provision

We were not able to obtain detailed records of additional educational support and/or on‐going speech‐language therapy provision. However, parents, teachers and special educational needs co‐ordinators were asked to report whether or not the child (a) was in receipt of a statement of special educational need, the legal document agreeing school placement and additional services required to meet a child's learning needs, (b) had moved from mainstream provision to a specialist school or resource base, and (c) had been referred to specialist speech‐language therapy services (Table 1). Details of speech‐language therapy provision could not be collected as these services were provided by the National Health Service. Such services could include combinations of assessment only, individualised feedback/intervention plan to school staff, training sessions for school staff, or direct intervention provided by therapists and/or trained assistants.

### Standardisation of core language and nonverbal measures

Sampling weights were constructed as the inverse of the predicted probability of a child being included in the study, so that when weighted, the estimates obtained from the sample are estimates for the whole population. Predicted probabilities of inclusion were estimated via two logistic models; the first logistic model was fitted in the entire population recruited to Phase 1 and included covariates predictive of inclusion due to study design. These were total number of pupils assessed per school and whether the child was identified as having high risk of language impairment based on CCC‐S teacher ratings (86th centile or above for sex and age group). The second logistic model was fitted only to children completing the second phase of the study. Covariates were tested in a stepwise elimination process. These were factors predictive of inclusion due to individual characteristics of the participants, such as sex, age group, IDACI rank score, English as an additional language and CCC‐S total raw score; and school‐level factors such number of pupils on role, percentage girls, percentage with identified special education needs and percentage receiving free school meals (a measure of school‐level deprivation). The final weights were a multiplication of the inverse of the predicted probabilities from the two models.

Given that many core language tests did not have current or valid UK standardisations, all language and nonverbal composites were standardised using the LMS method (Cole & Green, 1992). *Z*‐scores were calculated using a box‐cox (Box & Cox, 1964) type of transformation, whose parameters are estimated via penalised maximum likelihood. Moreover, the mathematical relationship between *z*‐scores and percentiles allows for the construction of smoothed centile curves across the entire distribution of a measure, similar to centile curves used in paediatric height and weight charts (G. Vamvakas, C.F. Norbury, S. Vitoratou, D. Gooch, & A. Pickles, under review).

Complete data on the language composites existed for 529/636 children for Year 1 and 499/529 for Year 3. No imputation was performed, but sampling weights take into account these missing observations. All available covariates that influence the ‘missingness’ indicator were used in order to maximise the likelihood of the data being missing at random.

### Testing lag

At Year 1, schools were randomly assigned to one of six testing blocks (blocks 1–6), which coincided with school half‐terms; approximately 100 children were assessed in each testing block. At Year 3, the order of testing blocks was reversed such that children seen in Block 6 at Year 1 were seen in Block 1 at Year 3. As a result, the lag between first and second assessment ranged from 15 to 27 months, creating a variable developmental window to observe language growth and thus allowing maximal benefit of a longitudinal design with two testing points.

### Statistical analysis

All statistical analyses were undertaken in Stata 14 (Stata Corporation, 2015). Language composite scores at both time points were analysed using a weighted, random intercepts growth model (mixed). Models were fitted to both raw and standardised language composite scores. Analysis of raw scores enables us to determine whether the slopes, representing increase in total score over time (or improvement in language in real terms), differ across the three language groups. For clarity, we report growth on the total language composite, which is an average of all six core language measures. Given that these tests are not scaled in the same way, growth models of individual language assessment raw scores are provided in supplemental materials (Tables S2 & S3). Where the pattern of change in individual tests differs from the composite, we explicitly discuss this in the main text; otherwise details of all individual tests are reported in supplemental materials. Analysis of *z*‐scores allows us to ask whether any positive increases in language raw scores over time are sufficient to narrow the gap between children with lower levels of language ability at intake and their typically developing peers.

## Results

### Demographic variables and descriptive statistics

Children with LD‐only were more likely to come from lower income neighbourhoods (IDACI rank scores) than TD peers, *t*(440) = 5.31, *p *<* *.001. Children with LD‐plus did not differ from TD peers or those with LD‐only with respect to neighbourhood disadvantage, *t*‐values 1.62 and 1.22 respectively, *p*s > .11. Both LD‐only and LD‐plus groups obtained higher teacher ratings of social, emotional and behavioural problems relative to TD peers, though children with LD‐plus were more likely to have clinically significant levels of behavioural difficulties (TD 10.24%, LD‐only 19.77%, LD‐plus 47.73%). By Year 3, children with LD‐plus were also more likely than peers to have statements of special educational need (50%), to be educated outside of mainstream classrooms (39.02%), and to have been referred for specialist speech‐language therapy assessment (84.09%), reflecting their more severe language impairments and pervasive developmental challenges. Specialist support for children with LD‐only was more variable, with fewer children qualifying for a statement (8.14%) or specialist provision (0%), and only half (54.65%) receiving referral to speech‐language therapy services.

### Growth in language skills

In these analyses, we excluded those children with a known clinical diagnosis who did not meet SCALES criteria for language disorder, due to the very small number of children in this category (*n* = 17). Language scores for the remaining children were centred at the mean age across the entire testing period (83 months). Unadjusted models estimated growth for both groups with Language Disorder (LD‐only; LD‐plus) relative to language growth in TD peers by including a group × age interaction term in the model. A significant interaction would indicate a difference in slope relative to the TD group. Subsequent adjusted models considered the influence of covariates individually and in interaction with age. Significant covariate × age interactions and marked changes to the coefficient of the interaction term would indicate that one or more covariate influences rate of language growth (slope).

**Figure 2:**
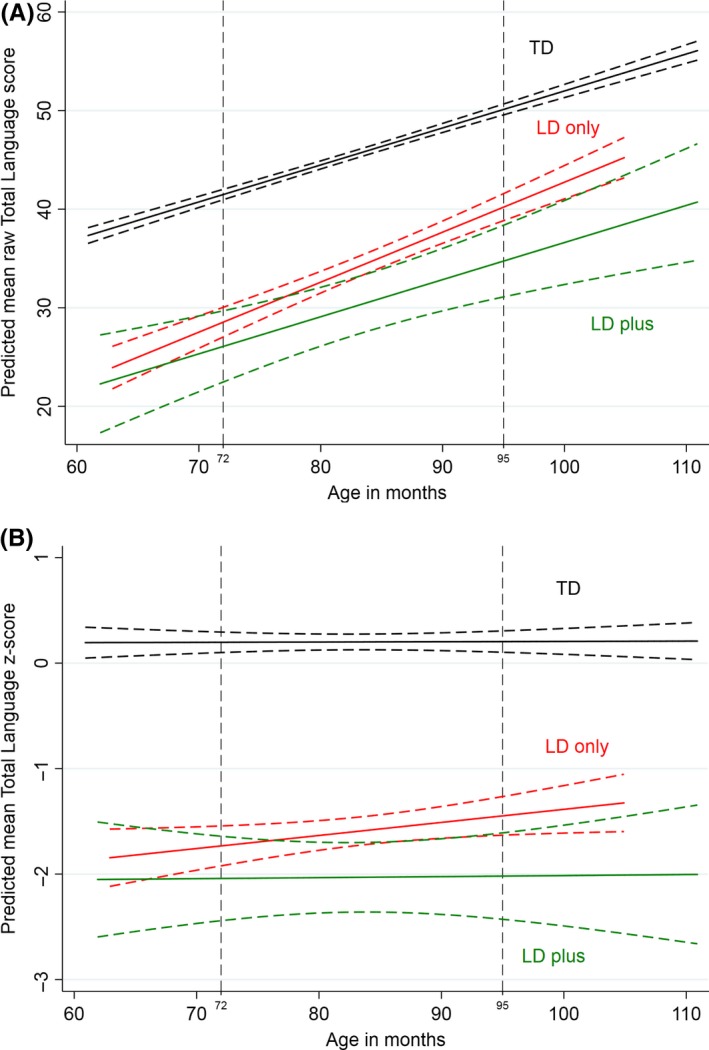
(A) Growth curve depicting change in raw scores on the total language composite from Year 1 to Year 3 for the typically developing (TD) children (top line), language disorder of unknown origin (LD‐only, middle line) and Language Disorder with known medical diagnosis/intellectual disability (LD‐plus). Curves for each group depict the means and the surrounding dashed lines the 95% confidence intervals. Vertical dashed lines indicate the mean age at Year 1 (left) and Year 3 (right). (B). Growth curve depicting change in *z*‐scores on the Total Language Composite from Year 1 to Year 3 for the Typically Developing (TD) children (top line), Language Disorder of unknown origin (LD‐only, middle line) and Language Disorder with known medical diagnosis/intellectual disability (LD‐plus). Curves for each group depict the means and the surrounding dashed lines the 95% confidence intervals. Vertical dashed lines indicate the mean age at Year 1 (left) and Year 3 (right)

For total language composite raw scores, the main effect of age was significant, indicating significant change in raw language scores over the three year period, increasing by 0.379 (*p* < .001) for each month increase in age (see Table 5). The group × age interaction was significant for the LD‐only group, β = .1021, *p* < .001. This indicates that the rate of increase was significantly greater for this group relative to TD peers. In contrast, the interaction term was not significant for children with LD ‐plus diagnosis, β = .0275, *p* = .579, indicating parallel rates of growth for these children relative to TD peers (Figure 2A). The first adjusted model included nonverbal IQ in Year 1, Total Difficulties scores at school entry, and IDACI rank score. Together, these three variables significantly improved model fit, Wald(8) = 3,860.54, *p* < .001 and each significantly predicted the language intercept. However, none of the covariate × age interaction terms was significant, nor did inclusion of these interaction terms affect the group × age coefficients. Thus, while these variables significantly predict a child's level of language ability, they do not significantly influence rate of language growth during this developmental period.

**Table 5:** Linear mixed effect models predicting growth in Total Language Composite raw scores from Year 1 and Year 3. Growth estimates (Group × Age interaction coefficients) are relative to the TD reference group. Model 1 = unadjusted, Model 2 = adjusted for individual covariates, Model 3 = Adjusted including interactions between covariate and age

	Model 1 β (95% CI)	Model 2 β (95% CI)	Model 3 β (95% CI)	Model 3 *p*‐value
Raw Scores Model				
Age	.3787 (0.362, 0.396)	.379 (0.3620, 0.396)	.375 (0.321, 0.428)	<.001
SDQ		−.708 (−1.242, −0.174)	−.704 (−1.239, −0.170)	.010
IDACI		.743 (0.251, 1.236)	.743 (0.251, 1.234)	.003
NVIQ		2.222 (1.805, 2.638)	2.223 (1.807, 2.638)	<.001
Group				
LD‐only	−10.729 (−11.787, −9.672)	−8.714 (−9.706, −7.723)	−8.713 (−9.704, −7.721)	<.001
LD+plus diagnosis	−17.032 (−19.178, −14.885)	−12.115 (−14.417, −9.813)	−12.113 (−14.414, −9.811)	<.001
Group × age				
LD‐only	.108 (0.051, 0.1650)	.109 (0.051, 0.166)	.111 (0.050, 0.171)	<.001
LD‐plus diagnosis	.029 (−0.082, 0.140)	.029 (−0.082, 0.140)	.029 (−0.097, 0.154)	.652
SDQ × age			.005 (−0.024, 0.035)	.716
IDACI × age			.001 (−0.020, 0.023)	.926
NVIQ × age			.002 (−0.017, 0.021)	.832
Constant	43.757 (43.286, 44.228)	42.606 (41.449, 43.764)	42.60 (41.451, 43.763)	<.001

The second set of models included Total Language Composite *z*‐score as the outcome variable, as this highlights the extent to which children with language disorder are narrowing the gap with peers. As can be seen in Figure 2B, there is no main effect of age, with flat growth in all three groups, β = .0007, *p* = .652. This is expected in the TD group, because *z*‐scores take account of age and therefore age is not predictive of outcome. However, the group × age interaction was not significant for either group with language disorder (Table 6). Notably, while there was limited growth in *z*‐scores, the LD‐plus group did not show evidence of a widening gap with peers during this period. Inclusion of the covariates significantly improved prediction of language intercept, but did not affect the group × age interaction co‐efficient. Furthermore, none of the covariate × age interactions were significant, all *p*‐values > .71. Thus, as with language raw scores, nonverbal IQ, SDQ Total Difficulties and IDACI rank scores did not influence rate of language change during these first years of school.

**Table 6:** Linear mixed effect models predicting growth in Total Language Composite *z*‐scores from Year 1 and Year 3. Growth estimates (Group × Age interaction coefficients) are relative to the TD group. Model 1 = unadjusted, Model 2 = adjusted for individual covariates, Model 3 = Adjusted including interactions between covariate and age

	Model 1 β (95% CI)	Model 2 β (95% CI)	Model 3 β (95% CI)	Model 3 *p*‐value
*z*‐scores model				
Age	.001 (−0.002, 0.004)	.001 (−0.002, 0.004)	−.002 (−0.011, 0.006)	.611
SDQ		−.127 (−0.202, −0.052)	−.128 (−0.202, −0.053)	.001
IDACI		.107 (0.035, 0.178)	.107 (0.036, 0.178)	.003
NVIQ		.365 (0.302, 0.428)	.365 (0.302, 0.427)	<.001
Group				
LD‐only	−1.597 (−1.727, −1.466)	−1.269 (−1.394, −1.146)	−1.269 (−1.393, −1.146)	<.001
LD+plus diagnosis	−2.268 (−2.479, −2.058)	−1.458 (−1.708, −1.208)	−1.459 (−1.709, −1.208)	<.001
Group × age				
LD‐only	.009 (−0.001, 0.020)	.009 (−0.001, 0.020)	.010 (−0.001, 0.021)	.069
LD+plus diagnosis	.004 (−0.011, 0.018)	.004 (−0.011, 0.018)	.005 (−0.013, 0.022)	.607
SDQ × age			−.001 (−0.006, 0.004)	.808
IDACI × age			.001 (−0.002, 0.005)	.496
NVIQ × age			−.001 (−0.004, 0.003)	.930
Constant	−.119 (−0.202, −0.036)	−.270 (−0.443, −0.097)	−.271 (−0.444, −0.098)	.002

With regard to individual language tests, children with LD‐only demonstrated slightly accelerated growth in raw scores on three of the six tests: Receptive Vocabulary, Narrative Comprehension, and Sentence Imitation (Tables S2 and S3). However, their rate of growth did not differ from LD‐plus peers on any task, apart from Sentence Imitation, where the difference in slopes between the LD‐only and the LD‐plus groups was significant (*p* = .003). One potential confound here is that the LD‐plus group includes a much higher proportion of children rated as having NPS at study intake (TD: 2.62%, LD‐only: 11.63%; LD‐plus: 38.64%), and many of these children were still challenged by complex, multiword utterances. Thus, differences between the LD‐only and LD‐plus groups could reflect lack of test sensitivity to increasing sentence length and complexity, due to the binary nature of response scoring. In addition, many children in the TD group are approaching ceiling on this task. Thus, ‘accelerated’ growth in the LD‐only group may reflect the fact that these children obtain scores in the middle of the distribution where there is more room to grow.

## Discussion

The SCALES cohort provides a unique opportunity to observe language trajectories of children with different language, cognitive, social and behavioural profiles relative to typically developing peers over the first four years of primary education. Our cohort included children with LD‐only, who had more variable nonverbal ability scores than previous epidemiological cohorts (Beitchman et al., 1996; Tomblin et al., 1997), but did not have intellectual disability or other known clinical syndromes when they started school. We considered their progress relative to children with LD‐plus, who experienced more severe language disorders that occurred in the context of a known clinical condition (such as autism spectrum disorder or Down syndrome) and/or intellectual disability. The most striking finding from our study is that these children with multiple developmental concerns demonstrated parallel rates of language growth relative to TD peers during this developmental period. Importantly, both groups of children with language disorder demonstrate improvement in absolute levels of language ability, and they maintain their relative standing within the cohort over this 3‐year period. Thus, our findings confirm and extend investigations demonstrating that *accelerated* growth in language for children with language disorder at school entry is at best rare, and in general, unlikely in sufficient numbers to substantially narrow the gap with typical peers in the first few years of school (Beitchman et al., 1996; Bornstein et al., 2016a; Conti‐Ramsden et al., 2012; Rice, 2012; Rice & Hoffman, 2015). Nevertheless, those with the most significant developmental challenges were not falling further behind, at least during this developmental window.

Our findings complement previous investigations in demonstrating impressive stability in language function after school entry, regardless of the child's initial level of language competence (Bornstein et al., 2016a, 2016b; Conti‐Ramsden et al., 2012; Tomblin et al., 2003; Pickles et al. 2014; Rice & Hoffman, 2015). Longitudinal stability of language has been consistently reported despite study differences in the combination of language assessments used, the population assessed, and access to specialist education or clinical services. Such stability does not suggest that children's language abilities are immutable to change; in this study as in previous reports, children across the ability range demonstrated improvement in real terms. Despite increase in raw scores however, children maintain their relative standing with peers. In this regard, the observed patterns of language growth resemble growth in other childhood characteristics such as height, where there exist considerable individual differences, steady increases over time, but limited change in rank order. Our study adds considerably to previous work by demonstrating that these patterns of growth are similar regardless of nonverbal ability, social, emotional and behavioural problems, socioeconomic status and/or additional clinical condition. Our study also indicates that diagnostic instability most likely reflects regression to the mean (Eadie et al. 2014; Zhang & Tomblin, 2003) and that those with ‘resolved’ language disorder likely maintain language performance at the boundaries of diagnostic cut‐offs and remain vulnerable to increasing language challenges as they get older (cf. Snowling, Duff, Nash, & Hulme, 2016).

Our study is unique in comparing language progress in children with language disorder of unknown origin, children with language disorder as part of an existing clinical condition, and typically developing peers. It is often reported that children with lower nonverbal cognitive abilities are more likely to demonstrate persistent language impairment, and slower rates of language growth (cf. Bishop & Edmundson, 1987). In SCALES, there is a complex relationship between nonverbal ability and language group status. Children with LD‐only had more variable nonverbal ability scores than in previous studies, and when language disorder was associated with a known clinical conditions and/or intellectual disability, children had, on average, significantly lower nonverbal ability, more severe language deficits and more pervasive social, emotional, behavioural and academic deficits. Thus, nonverbal ability did predict initial variance in language scores, but it did not influence rate of change on the omnibus language measure.

Children with LD‐plus did differ from LD‐only on the measure of expressive grammar (cf. Rice, 2015). This likely reflects measurement issues and the sensitivity of this instrument to detect changes in expressive grammar given the binary scoring criteria we employed. Another caveat is the greater variation within the LD‐plus group, arising from relatively small numbers and heterogeneous clinical conditions. With further testing periods, it may be possible to identify latent growth profiles irrespective of pre‐existing diagnoses. This could further elucidate factors that enable children to maintain a steady rate of language growth despite numerous developmental challenges. Nevertheless, similar rates of growth on most measures suggest that similar processes underscore language growth, despite different initial starting states (Bornstein et al., 2016a; Rice, 2012). The extent to which these processes are amenable to change is a needed focus of future research.

Neither group with language disorder demonstrated sufficient language progress to substantially narrow the gap with TD peers when age‐adjusted *z*‐scores were considered. This is somewhat surprising, giving the significant difference in growth observed for the LD‐only group when using raw scores. We considered whether growth in raw scores may be unduly influenced by different patterns of growth in one or more individual language tests (cf. Rice, 2012, see supplemental materials). Differences in slope between the LD‐only and TD group were observed on three of the six language tests (receptive vocabulary, expressive grammar and narrative comprehension), which at least for grammar and narrative comprehension may at least partially reflect the fact that TD children were starting to reach ceiling on these measures. Thus, improvement was not evident on all aspects of language and not sufficient on any one measure to affect the rank order of participants within the population.

The study was not designed to address education or clinical provision, but it is worth noting that all groups show improvement in real terms and neither the LD‐only or LD‐plus groups demonstrated plateau or deceleration of language growth, at least in the first few years of primary school. Children with LD‐plus were much more likely to be receiving specialist educational support than peers with LD‐only and most had been referred to speech‐language therapy services. However, the current study cannot determine whether it is this provision that supports the steady rate of language growth in these children. Given that lower nonverbal ability is often used as an exclusion criteria for specialist language intervention (Dockrell, Lindsay, Letchford, Mackie, 2006), intervention studies that explicitly test the influence of nonverbal ability on response to treatment are urgently needed. Follow‐up of this group will be particularly informative for elucidating how early multiple developmental challenges may impact later language learning. It may be anticipated that rate of growth for children with LD‐plus will slow as typical learning opportunities and environmental input are altered by the experience of having profound language impairment.

It has been suggested that the degree of stability in language function after the age of five or six reflects diminishing neural plasticity to respond to and learn from enhanced environmental language input (Bornstein et al., 2016b; Pickles et al. 2014). The current findings provide clear evidence that children with language disorders can learn and acquire new language forms during the early school years, indicating that the language system remains responsive to input during this developmental period. Universal, high quality education may contribute to the uniform rates of growth observed in this, and other studies from western, industrialised nations. However, the finding that children with multiple developmental challenges demonstrate the same rate of growth as TD peers raises interesting questions about the extent to which ‘rate’ of language learning is malleable. To ‘narrow the gap’ with TD peers requires children with biological and/or environmental language learning challenges to develop at a faster rate than TD peers in response to enhanced input, such as intervention. It is currently unknown whether this is possible, whether there is an optimal developmental window in which rate may be more amenable to change, or what intensity or scope of intervention is required to alter a developmental language trajectory. Longitudinal studies appear to suggest that typical provisions may prevent vulnerable children from falling further behind, but are not sufficient to alter developmental language trajectories for most children. This should not preclude future trials from testing what is possible if more intensive, or more specifically targeted interventions are provided. Another possibility may be that moving children with language disorder into the average range of language function may not be a realistic goal of therapy services. Given the substantially lower starting point for children with LD‐plus, significant changes in raw score at the tail of the distribution would not necessarily impact standard scores. Instead, maximising language skills required to access the curriculum and sustain meaningful social relationships may be a worthy goal of specialist clinical and education services.

### Strengths and limitations

Our study is unique in that it utilises a population cohort that includes children with a wide range of language abilities and developmental profiles. We minimised measurement variation by using exactly the same language measures at both testing points, which were co‐standardised. The study is limited, however, by the fact that we were only able to assess children at two time points. The variable period between assessments increased the range of development from 15 to 27 months, allowing us to model growth over a longer developmental period than would have been possible using more traditional longitudinal designs. Nevertheless, we were only able to model linear growth and it is possible that with additional observations, we might detect different patterns of growth such as rapid improvement followed by plateau or decline (cf. Rice & Hoffman, 2015). We also did not include measures of literacy in our predictors of language growth. Literacy is an important avenue for language learning in school‐aged children, and avid readers demonstrate accelerated growth in vocabulary over time (Duff, Tomblin & Catts, 2015). Thus, literacy may further elucidate potential progress in children with language deficits. Our study is also limited in that we only assessed children after school entry, and thus do not have information about language growth for these children during the preschool years. As this was a school‐based study, we have limited information about the child's home environment or family history of language disorder. While measures of maternal language and education have not been found to relate to language stability in previous studies (Bornstein et al., 2016a, 2016b), it is possible that genetic risk factors may contribute to rate of language growth. Finally, more detailed information about the clinical services these children received, including the content and consistency of speech‐language therapy input, could further elucidate the potential for language change in this cohort.

## Conclusion

The current findings demonstrate stability in language across the first four years of school, characterised by slow and steady growth for children with varying degrees of language disorder and co‐occurring developmental concerns. While there was limited evidence that children with language disorder have narrowed the gap with typical peers in the early school years, children with multiple developmental challenges were not falling further behind. Growth in language was not modulated by nonverbal abilities, symptoms of social, emotional or behavioural problems, additional clinical diagnoses or socioeconomic factors. These findings raise theoretically interesting questions about the extent to which rate of language growth is malleable in children with neurodevelopmental disorders.

**Key points**

- Numerous studies highlight stability in individual differences in language ability from school entry.
- Children with ‘specific’ developmental language disorder demonstrate parallel rates of growth relative to typically developing peers.
- Children with ‘specific’ developmental language disorder demonstrate accelerated language growth on some language measures relative to typical peers, but this is insufficient to close the gap when age‐adjusted *z*‐scores are considered.
- Children with language disorders and additional clinical diagnoses and/or intellectual impairment demonstrated parallel rates of growth relative to typically developing peers.
- There was no evidence that children with language disorders and additional clinical diagnoses were falling further behind in language function during the first four years of school.
- Nonverbal cognitive deficits and/or social, emotional and behavioural deficits should not preclude access to interventions aimed at developing language skills, though the impact of these factors on response to treatment requires systematic investigation.
- ‘Narrowing the gap’ or ‘normalising’ language function may not be a realistic goal of intervention services for school‐aged children; enabling children to learn language necessary for social and academic participation is a key priorityT/S please query authors to check and verify changes.

## Supporting information


**Table S1.** Estimated means (95% confidence intervals) using probability weights for key measures by group (based at Year 1 group classification) when tested in Year 1 of school and again at Year 3.
**Table S2.** Linear mixed effects models for expressive language indices with raw scores as the dependent variable.
**Table S3.** Linear mixed effects models for receptive language indices with raw scores as the dependent variable.
