# Supplementary material for: Language growth in children with heterogeneous language disorders: a population study
Source: J Child Psychol Psychiatry. 2017 Sep 18;58(10):1092–105. doi: 10.1111/jcpp.12793 (PMC5639364; doi:10.1111/jcpp.12793)
Supplement: Supplementary file 1 — Table S1. Estimated means (95% confidence intervals) using probability weights for key measures by group (based at Year 1 group classification) when tested in Year 1 of school and again at Year 3. Table S2. Linear mixed effects models for expressive language indices with raw scores as the dependent variable. Table S3. Linear mixed effects models for receptive language indices with raw scores as the dependent variable. [file JCPP-58-1092-s001.docx]

**Online Supporting Information for: Language growth in children with heterogeneous language disorders: a population study – by Norbury et al.**

Table S1. Estimated means [95% confidence intervals] using probability weights for key measures by group (based at Year 1 group classification) when tested in Year 1 of school and again at Year 3. N refers to the number of participants assessed in each group prior to adjustment by probability weights. All measures are reported as raw language scores.

|  | Typical | Develop | Language  (no known | Disorder only  Diagnosis) | Language  (known diagnosis/ | Disorder plus  intellectual  disability) |
| --- | --- | --- | --- | --- | --- | --- |
| measure | Year 1  N = 381 | Year 3  N = 359 | Year 1  N = 86 | Year 3  N = 82 | Year 1  N = 45 | Year 3  N = 42 |
| Age (months) | 71.70  [71.06, 72.34] | 95.27  [94.67, 95.86] | 71.88  [70.63, 73.13] | 95.96  [94.45, 97.47] | 73.55  [72.33, 74.78] | 95.22  [92.79, 97.64] |
| ExpressiveVocab | 83.48  [81.96, 85.01] | 100.28  [98.98, 101.59] | 61.43  [57.60, 65.25] | 81.30  [77.16, 85.44] | 57.80  [54.21, 61.39] | 75.44  [67.55,  83.32] |
| Receptive Vocab | 85.71  [84.25, 87.19] | 105.46  [104.01, 106.92] | 65.43  [61.06, 69.80] | 91.32  [87.92, 94.73] | 64.00  [57.42, 70.58] | 81.16  [74.41, 87.92] |
| Expressive Grammar | 20.81  [19.99, 21.64] | 25.44  [24.84, 26.04] | 7.84  [5.77, 9.90] | 15.81  [13.43, 18.20] | 8.52  [1.69, 15.34] | 13.69  [6.00, 21.38] |
| Receptive Grammar | 28.31  [27.61, 29.02] | 33.75  [33.30,  34.19] | 20.02  [18.36, 21.68] | 27.29  [25.57,  29.01] | 14.61  [11.22, 18.00] | 21.44  [16.26, 26.62] |
| Expressive Narrative | 13.57  [13.04, 14.11] | 18.25  [17.74, 18.75] | 7.37  [5.73, 9.01] | 13.40  [11.48, 15.32] | 5.40  [4.71, 6.09] | 11.07  [9.28, 12.85] |
| Receptive Narrative | 15.39  [14.91, 15.88] | 18.63  [18.28, 18.99] | 8.87  [7.24, 10.49] | 14.38  [13.08, 15.68] | 4.69  [3.10, 6.29] | 10.56  [8.85, 12.28] |
| Total language composite (raw) | 41.22  [40.52, 41.92] | 50.30  [49.75, 50.85] | 28.49  [26.75, 30.24] | 40.58  [38.68, 42.49] | 25.84  [23.00, 28.67] | 35.56  [30.75, 40.37] |

Vocabulary = Expressive One Word Picture Vocabulary Test, range 0 – 136; Grammar = School-aged Sentence Imitation Task-32, range 0-32; Narrative = Assessment of Comprehension and Express 6-11 Narrative sub-test, range 0-35.

Receptive Vocabulary = Receptive One Word Picture Vocabulary Test, range 0 – 153; Grammar = Test for Reception of Grammar- short form, range 0-40; Narrative = Bespoke comprehension questions for Assessment of Comprehension and Express 6-11 Narrative, range 0-24.

Table S2. Linear mixed effects models for expressive language indices with raw scores as the dependent variable. These are final, adjusted models including covariates of non-verbal IQ, IDACI rank scores (a measure of SES) and SDQ Total Difficulties raw score (a measure of social, emotional and behavioural deficits).

| **Raw Scores** | **Model 1**  **vocabulary**  **β [95% CI]** | **p-value** | **Model 2**  **grammar**  **β [95% CI]** | **p-value** | **Model 3**  **narrative**  **β [95% CI]** | **p-value** |
| --- | --- | --- | --- | --- | --- | --- |
| **Wald(11)** | 1695.34 | <.001 | 1572.68 | <.001 | 993.85 | <.001 |
| **Age** | 0.73  [0.58, 0.87] | <.001 | 0.23  [0.15, 0.31] | <.001 | 0.14  [0.08, 0.21] | <.001 |
| **Co-variates** |  |  |  |  |  |  |
| SDQ | -0.16  [-1.49, 1.17] | .814 | -1.15  [-1.87, -0.44] | .002 | -0.53  [-0.89, -0.17] | .004 |
| IDACI | 2.18  [1.04, 3.33] | <.001 | 0.74  [0.08, 1.41] | .028 | -0.01  [-0.36, 0.34] | .974 |
| NVIQ | 4.21  [3.28, 5.13] | <.001 | 1.67  [1.18, 2.16] | <.001 | 1.13  [0.85, 1.41] | <.001 |
| **Group** |  |  |  |  |  |  |
| LD-only | -15.94  [-18.49,-13,39] | <.001 | -9.16  [-10.44, -7.88] | <.001 | -3.75  [-4.45, -3.05] | <.001 |
| LD-plus diagnosis | -18.60  [-23.64, -13.56] | <.001 | -10.59  [-13.25, -7.94] | <.001 | -5.81  [-6.97, -4.65] | <.001 |
| **Group * age** |  |  |  |  |  |  |
| LD-only vs TD | 0.11  [-0.05, 0.26] | .172 | 0.10  [0.03, 0.17] | .005 | 0.06  [-0.05, 0.17] | .292 |
| LD-plus diagnosis vs TD | 0.05  [-0.29, 0.39] | .776 | -0.06  [-0.16, 0.04] | .214 | 0.01  [-0.10, 0.11] | .918 |
| LD-plus diagnosis vs LD-only | -0.06  [-0.40, 0.29] | .748 | -0.16 [-0.27, -0.06] | .003 | -0.05 [-0.18, 0.04] | .410 |
| **Co-variates * age** |  |  |  |  |  |  |
| SDQ | 0.04  [-0.03, 0.10] | .283 | 0.015  [-0.019, 0.050] | .383 | -0.01  [-0.03, 0.04] | .755 |
| IDACI | -0.02  [-0.08, 0.04] | .583 | -0.01  [-0.04, 0.02] | .372 | 0.02  [-0.01, 0.05] | .096 |
| NVIQ | 0.003  [-0.049, 0.055] | .916 | -0.023  [-0.044, -0.002] | .030 | -0.02  [-0.04, 0.01] | .236 |
| **Constant** | 83.93  [81.18, 86.67] | <.001 | 20.05  [18.41, 21.69] | <.001 | 15.47  [14.63, 16.32] | <.001 |

Table S3. Linear mixed effects models for receptive language indices with raw scores as the dependent variable. These are final, adjusted models including covariates of non-verbal IQ, IDACI rank scores (a measure of SES) and SDQ Total Difficulties score (a measure of social, emotional and behavioural deficits).

| **Raw Scores** | **Model 1**  **vocabulary**  **β [95% CI]** | **p-value** | **Model 2**  **grammar**  **β [95% CI]** | **p-value** | **Model 3**  **narrative**  **β [95% CI]** | **p-value** |
| --- | --- | --- | --- | --- | --- | --- |
| **Wald(11)** | 1653.57 | <.001 | 1311.17 | <.001 | 864.25 | <.001 |
|  |  |  |  |  |  |  |
| **Age** | 0.81  [0.62, 1.01] | <.001 | 0.22  [0.15, 0.29] | <.001 | 0.12  [0.06, 0.18] | .001 |
| **Co-variates** |  |  |  |  |  |  |
| SDQ | -1.38  [-2.46, -0.29] | .013 | -0.64  [-1.22., -0.05] | .034 | -0.44  [-0.81, -0.08] | .018 |
| IDACI | 1.27  [0.28, 2.27] | .012 | 0.26  [-0.28, 0.79] | .346 | -0.03  [-0.35, 0.29] | .869 |
| NVIQ | 3.71  [2.79, 4.62] | <.001 | 1.76  [1.34, 2.17] | <.001 | 0.98  [0.70, 1.26] | <.001 |
| **Group** |  |  |  |  |  |  |
| LD-only | -12.51  [-14.76, -10.26] | <.001 | -6.83  [-7.94, -5.71] | <.001 | -4.28  [-4.95, -3.61] | <.001 |
| LD-plus diagnosis | -17.95  [-22.09, -13.81] | <.001 | -11.67  [-14.27, -9.08] | <.001 | -7.49  [-8.83, -6.15] | <.001 |
| **Group * age** |  |  |  |  |  |  |
| LD-only vs TD | 0.26  [0.10, 0.41] | .001 | 0.06  [-0.01, 0.14] | .086 | 0.078  [0.004, 0.152] | .038 |
| LD-plus diagnosis vs TD | 0.06  [-0.14, 0.27] | .551 | 0.03  [-0.13, 0.18] | .723 | 0.09  [-0.05, 0.23] | .217 |
| LD-plus diagnosis vs LD-only | -0.19 [-0.41, 0.02] | .079 | -0.04 [-0.20, 0.13] | .669 | 0.01 [-0.13, 0.15] | .905 |
| **Co-variates * age** |  |  |  |  |  |  |
| SDQ | -0.004  [-0.10, 0.09] | .935 | -0.01  [-0.06, 0.03] | .629 | -0.01  [-0.04, 0.03] | .651 |
| IDACI | -0.0001  [-0.0762, 0.0759] | .997 | 0.01  [-0.02, 0.03] | .710 | 0.01  [-0.02, 0.04] | .598 |
| NVIQ | 0.073  [0.006, 0.140] | .032 | -0.01  [-0.04, 0.02] | .484 | -0.01  [-0.04, 0.01] | .217 |
| **Constant** | 90.30  [87.99, 92.61] | <.001 | 29.64  [28.38,30.91] | <.001 | 16.47  [15.68, 17.26] | <.001 |

As can be seen in Tables S2 and S3, three tests yielded accelerated rates of growth in the LD-only group: receptive vocabulary, narrative comprehension, and sentence recall. In both receptive vocabulary and sentence repetition, the age x NVIQ interaction was also significant.

Our findings are largely consistent with Rice (2012). Rice measured rate of language growth between ages of 2 and eight years in children with ‘specific language impairment’ (defined as obtaining scores -1SD below the normative mean on an omnibus test of language in the context of non-verbal IQ scores of 85 or greater) and typically developing peers. The pattern of language growth varied depending on the measure of language included; vocabulary comprehension was characterised by largely linear growth and rapid improvement over this developmental period, while a measure of morphosyntax was characterised by rapid growth reaching a natural asymptote in the early school years when typical children reach mastery. Children with SLI also mastered this form, but took on average two years longer to reach that level of language maturity.

The current paper extends similar findings to children with more significant language and cognitive deficits, at least on measures of receptive vocabulary. We did find accelerated growth for children with LD-only in this narrower developmental window; however, children with LD-plus, who had more severe and pervasive deficits, followed a parallel trajectory to that of TD peers and did not differ significantly from peers with LD-only.

A different pattern was observed for the sentence recall measure (an index of expressive grammar). Here, accelerated growth was again seen in the LD-only group, and the difference in slopes between the LD-only and the LD-plus groups was significant (p = .003). One potential confound here is that the LD-plus group includes a much higher proportion of children rated as having No Phrase Speech (NPS) at reception (TD: 2.62%, LD-only: 11.63%; LD-plus: 38.64%), and many of these children were still challenged by complex, multi-word utterances. Thus, differences between the LD-only and LD-plus groups could reflect lack of test sensitivity to increasing sentence length and complexity, due to the binary nature of response scoring. In addition, many children in the TD group are approaching ceiling on this task. Thus, ‘accelerated’ growth in the LD-only group may reflect the fact that these children obtain scores in the middle of the distribution where there is more room to grow.

Rice (2012) argues that parallel growth trajectories in her cohort suggests similar mechanisms of timing and maturation contribute to language growth in both SLI and typical development. Additional follow-up of the SCALES cohort will determine whether these mechanisms persist over a longer developmental period and extend to children with multiple developmental challenges.
